# 3D Bioprinting and the Future of Surgery

**DOI:** 10.3389/fsurg.2020.609836

**Published:** 2020-11-27

**Authors:** Thomas H. Jovic, Emman J. Combellack, Zita M. Jessop, Iain S. Whitaker

**Affiliations:** ^1^Reconstructive Surgery and Regenerative Medicine Research Group, Swansea University, Swansea, United Kingdom; ^2^Welsh Centre for Burns and Plastic Surgery, Morriston Hospital, Swansea, United Kingdom

**Keywords:** 3D printing, transplantation, biotechnology, bioprinting, reconstruction

## Abstract

**Introduction:** The disciplines of 3D bioprinting and surgery have witnessed incremental transformations over the last century. 3D bioprinting is a convergence of biology and engineering technologies, mirroring the clinical need to produce viable biological tissue through advancements in printing, regenerative medicine and materials science. To outline the current and future challenges of 3D bioprinting technology in surgery.

**Methods:** A comprehensive literature search was undertaken using the MEDLINE, EMBASE and Google Scholar databases between 2000 and 2019. A narrative synthesis of the resulting literature was produced to discuss 3D bioprinting, current and future challenges, the role in personalized medicine and transplantation surgery and the global 3D bioprinting market.

**Results:** The next 20 years will see the advent of bioprinted implants for surgical use, however the path to clinical incorporation will be fraught with an array of ethical, regulatory and technical challenges of which each must be surmounted. Previous clinical cases where regulatory processes have been bypassed have led to poor outcomes and controversy. Speculated roles of 3D bioprinting in surgery include the production of *de novo* organs for transplantation and use of autologous cellular material for personalized medicine. The promise of these technologies has sparked an industrial revolution, leading to an exponential growth of the 3D bioprinting market worth billions of dollars.

**Conclusion:** Effective translation requires the input of scientists, engineers, clinicians, and regulatory bodies: there is a need for a collaborative effort to translate this impactful technology into a real-world healthcare setting and potentially transform the future of surgery.

## Introduction

The advent of three-dimensional (3D) printing has evoked a global industrial revolution, garnering the attention of the public and media in the process. Despite having its roots in the automotive, packaging and architectural domains (1), major developments in 3D printing technology have born witness to an expanded role of printing technologies, spanning into healthcare research and prompting the development of numerous medical devices, models and prosthetics.

Surgery too has witnessed incremental transformations over the past century, with the introduction of microsurgery, transplantation and robotics augmenting the array of treatments available for patients. As the scope and complexity of surgical interventions broadens so too does the need to adequately plan and prepare for surgery. Furthermore, many procedures, particularly in reconstructive and transplantation surgery remain hindered by the availability of donor tissues and organs, the morbidity associated with tissue harvest and the potential complications related to immunosuppression (2, 3). 3D printing software can be used to extract digital data from patient images such as computed tomography, magnetic resonance imaging or laser scanning to yield custom-made and personalized constructs for surgical planning and implantation (1). In particular, the incorporation of a biological component would transform this established technology, with the potential to revolutionize personalized healthcare through the advent of autologous living implants akin to the patient's own tissue.

3D printing holds incredible potential for the future of surgery, as acknowledged by the Royal College of Surgeons in the Commission on the Future of Surgery (4). The biological applications of 3D printing technology, or “bioprinting,” traverse the disciplines of human biology, materials science and mechanical engineering, and incorporates this into clinical practice to yield novel and personalized surgical options for patients (5, 6). Successful implementation could lead to a paradigm shift in surgical outcomes, with the potential to obviate the need for donor organs for transplantation surgery and offering the restoration of form and function without painful and destructive donor sites (7). Throughout the course of this review article we aim to identify the key roles this technology may play in the future of surgery and explore the pivotal considerations and challenges that remain to be addressed prior to the integration of three-dimensional printing and bioprinting into mainstream surgical practice.

## 3D Bioprinting in Surgery

### Addressing the Current and Future Challenges to Translation

#### The Promise of 3D Bioprinting

The introduction of 3D printing into surgical practice is already underway. An example of successful integration is the ability to 3D print customized titanium prosthetics which has revolutionized personalized maxillofacial surgery in the UK (8), and the ability to emulate the success of this technology in 3D printing tissue holds the potential to revolutionize transplantation surgery and reconstructive surgery (9).

**Figure 1:**
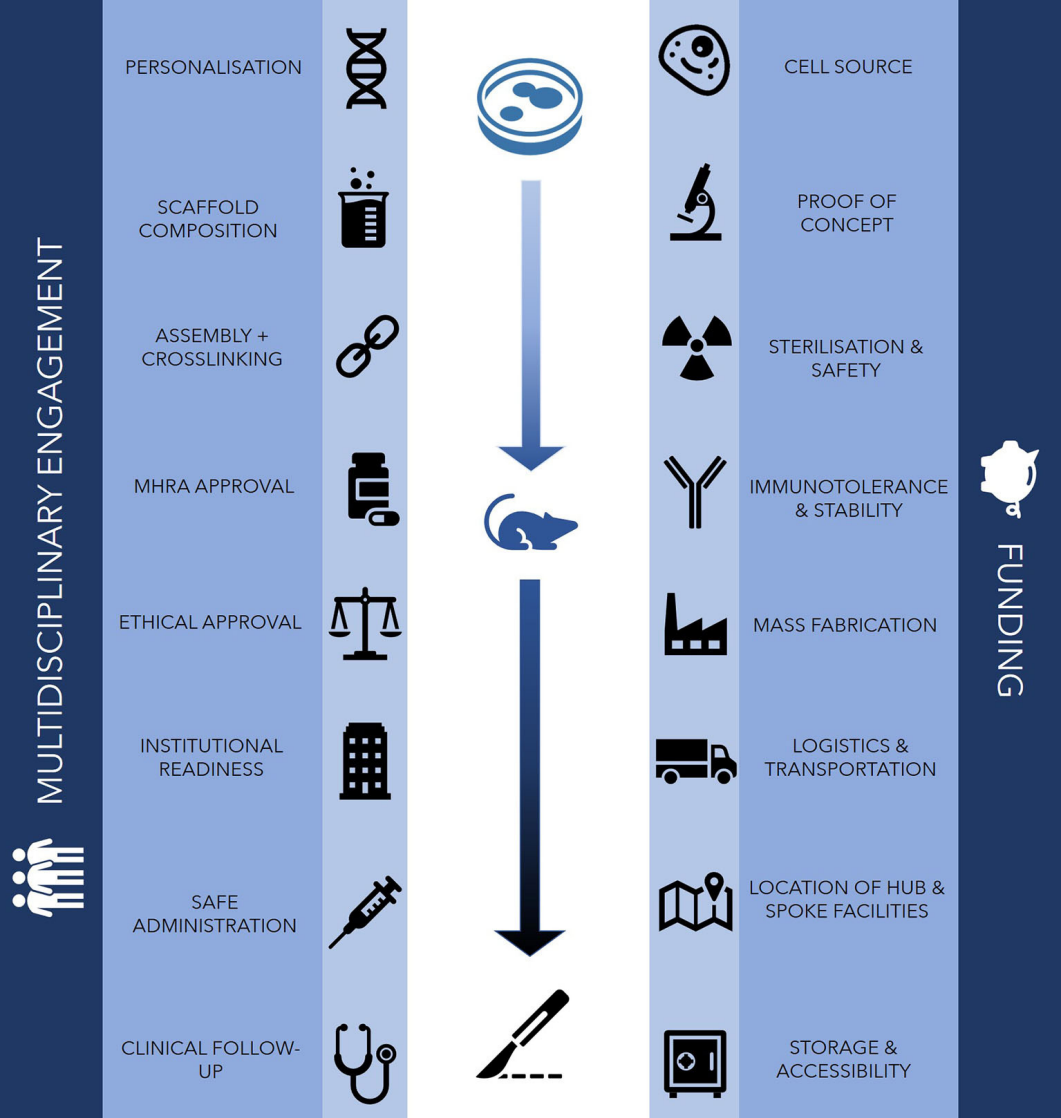
Challenges for clinical translation in 3D bioprinting. The main challenges to clinically translating bioprinting technology traverse *in vitro, in vivo* and clinical domains, requiring the support of financial investment, a robust logistical network and engagement from multidisciplinary professionals.

It is the promise of a biological component in technologies such as bioprinting that presents the most significant challenges to 3D printing in surgery. There have been a number of recent reports that have raised concerns regarding the adoption of regenerative medical interventions such as 3D bioprinting into mainstream clinical practice (10). The process of bioprinting requires cells, bioinks and bioprinters, each of which presents biological, technical and ethical challenges and uncertainty regarding clinical effectiveness and cost-effectiveness (11). As such, the translation of 3D bioprinting into mainstream clinical practice will be fraught with significant challenges (Figure 1).

#### Challenges of Cell Source Selection

Within the healthcare setting itself, the origins of both cell sources and bioink materials may spark further debate. Firstly, cells used to create simple tissue structures such as heart valves could feasibly be derived from either animals or humans, as with the porcine valves currently used in clinical practice. Animal sources are likely to enable greater mass production of tissue for surgical use but consist of allogenic material with a risk of disease xenotransmission (12). In contrast, human sources offer greater biocompatibility and the opportunity for personalisation, but their use is likely to be fraught with tighter regulation, lengthier production times and higher costs. Many donor related ethical concerns could be bypassed by the use of autologous cell sources, however the accessibility of certain cell types and the presence of genetic diseases may cloud the ethical and regulatory aspects of autologous cell sourcing (13). In addition, due to limited human trials of successful clinical translation of tissue engineered constructs at present, there remains an element of unpredictability regarding how autologous cells will behave. The biological component of implants make integration and interactions more unpredictable when inserted into hosts than currently used stents, pacemakers and artificial joints. Differences in patient's genotypes will affect processes such as cell migration, post-printing phenotype, oncogenic potential (particularly in immortalized cell populations) and dysregulated differentiation, such as fat derived stem cells producing ectopic bone for example (14, 15). Teratoma formation and the recurrence or potentiation of malignancy from the use of stem cells remains a significant scientific concern: a first in human trial of induced pluripotent stem cells in Japan was ceased due to the genomic mutations that developed (16, 17).

#### Challenges of Biological Ink (Bioink) Selection

Irrespective of cell sources, the materials selected for bioink production must be biocompatible before being considered for use in humans. The immunogenicity and toxicity of bioinks will necessitate further investigation prior to human trials (18). Many materials are derived from non-human organisms such as alginate from seaweed and gelatin from porcine material. The foreign nature of these components risk immunogenicity, inflammation and infection (12, 19). The production of degradable biomaterials has attracted significant research interest due to their potential to create scaffolds that resorb, as new tissue forms to replace it (18). Further research is also needed to investigate the risk of toxicity as by-products are released into bloodstream, and undergo renal or hepatic clearance (20). The bioprinting process itself, in particular for extrusion based bioprinting, may exert shear forces on cells. Mechanotransduction pathways may disrupt cell behavior and direct stem cells down certain, potentially undesired lineages (21). Furthermore, many hydrogel materials used as bioinks are required to be crosslinked post-printing to maintain their 3D shape. Crosslinking often requires chemical, thermal or enzymatic catalysis which may be cytotoxic or induce DNA damage, an example of which is the use of ultraviolet light as a photopolymerisation agent (22). For many of these crosslinking processes, the genotoxic effects of free radicals and ultraviolet irradiation on DNA damage may not be immediately apparent (22).

#### Regulatory Challenges

Regulation of these products is another challenge. The high degree of personalisation in construct shape and genetic material renders bioprinted tissue a “custom made device.” The inclusion of biological material complicates the picture, and governing bodies worldwide such as the FDA are failing to keep abreast of the rapidly developing field of bioprinting, with currently unclear guidance and regulations for such technology (13). The challenge with regenerative medicine and tissue engineered technologies and their components are their classification and ultimate regulation in all facets of design, production, handing. The seemingly endless innovation and advancement of these technologies illustrates the intersection of a number of different pathways covering a broad taxonomy of perceived utility, cost effectiveness and biography (11). The classification of complex and novel regenerative and gene medicinal products was expanded to include tissue engineered constructs which sought to define Advanced therapy medicinal products (ATMPs) (23). This class of innovative therapies represents a novel group of therapeutic agents with significant differences to those therapies currently licensed and available on the market (24). In an effort to standardize market availability within the European Union (EU) the European Commission (EC) established the ATMP Regulation (EC 1394/2007) alongside directive 2009/120 which created definitions for these novel technologies alongside marketing authorization (MA) guidance (25). As one of the four product types covered by the ATMP umbrella, Tissue engineered products (TEPs) have seen a slow progression over the last 10 years, with relatively few transitioning from concept to patient application. The complexity of UK and EU regulation, coupled with challenges at a regional level with safety, scalability and reliable production has posed a number of challenges for regulators and applicants alike. The majority of these innovations are being produced within academic institutions rather than commercial enterprises (26). The challenges therefore must be addressed by clearer communication between regulatory bodies and organizations seeking to produce and market TEPs for clinical use. The EU regulatory committees recognize the challenges posed by the complex nature of these novel technologies and need for development of bespoke guidance as new challenges arise. In the UK, regulators such as the MHRA should be engaged early to facilitate the development of processes and pathways which will ultimately meet standards required to scale tissue engineered constructs for both clinical trials and commercial manufacture. Outside the healthcare setting, incremental advancements in three-dimensional printing technology are yielding more affordable and compact printing systems at an astonishing rate. This rapid evolution indicates three dimensional printers may eventually become household items, much like conventional inkjet printers (27). With these ambitious aspirations however, come fears of the power of such technology being widely accessible. Concerns of unregulated and “DIY” home use may facilitate bioterrorism (28) and unregulated surgical practices, much like the current epidemic of unregulated injectables such as botulinum toxin and fillers.

#### Ethical Challenges

The design of clinical trials will also prove challenging: it would be unethical to trial tissue engineered organ transplantation on healthy volunteers, and the use of patient specific cell populations mean that the patient themselves would need to act as their own control, introducing a high degree of heterogeneity when attempting to assess treatment efficacy (13). This could be particularly problematic when interpreting favorable results from clinical trial patients: how much of the effect is the patient's inherent response to treatment and how much is attributable to the bioprinted product itself? A valid and comprehensive means of evaluating the effect of bioprinted interventions need to be formally defined prior to commencing any clinical trials of value in this area. Indeed, the only trials of tissue engineered constructs to date have been in patients with terminal disease, where such “last resort” options are often considered “more ethical,” despite the uncertainty of complications. Examples include the use of skeletonized trachea from cadaveric sources, seeded with patient mesenchymal stem cells for use in surgery (29). In these instances, the key to acquiring ethical approval was in the portrayal of the patients' clinical urgency. Describing the trial of a tissue engineered trachea as the last resort option and as a final chance at a lifesaving intervention facilitated the acquisition of ethical approval to implement the treatment in patients (2, 30). This approach was effective in driving an incremental step in translational bioengineering but is a shortcut that presents significant limitations.

The caveat to such advancements is the potential to generate uncontrolled and unethical practice. This is particularly starkly demonstrated by the Macchiarini scandal, where the outcomes of the synthetic trachea implantation were falsely augmented (31). Indeed the inadequacy of preclinical evidence in this instance, reiterates the importance of a robust foundation of scientific and clinical validity prior to clinical implementation (3). Other challenges in designing clinical trials include the fact that patients cannot withdraw post-implantation, and consent for trial inclusion is challenging where the extent of complications is uncertain. A perceived benefit of three dimensional structures is that at least a degree of reversibility exists in their ability to be excised if problematic, whereas injectable stem cell and gene based therapies may prove extremely challenging, if not impossible, to reverse (13).

#### Technological and Institutional Readiness

**Figure 2:**
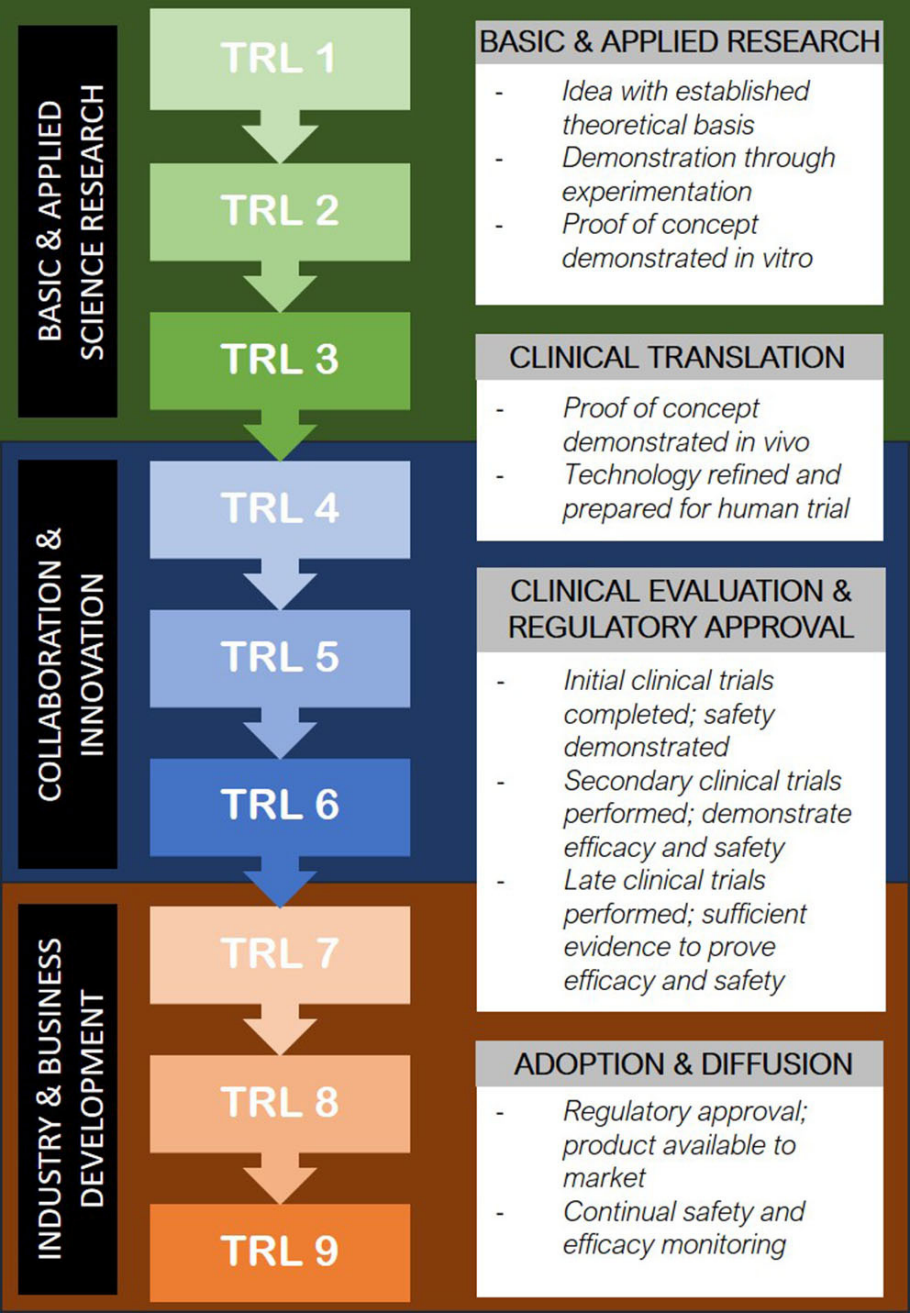
Translation of emerging medical technology into clinical use through the Technology Readiness Level model (32).

Should bioprinted technologies surpass the obstacles of clinical proof of concept, widespread uptake of the technology presents a further set of significant challenges. The pathway from conception to implementation in patients can be mirrored through the concept of the “Technology Readiness Scale” (32) (Figure 2). In the field of bioprinting, much of the current research exists in the TRL1-4 stages; *in vitro* experimentation with the optimisation of scaffold and cell source combinations, bioinks and 3D printing methods and construct analysis (33). There are a handful of groups worldwide who report the use of 3D bioprinted cartilage, bone, skin and vessel constructs in animal models (33) and occasional cases of 3D printing technology being used to make biological constructs in humans (34, 35). In addition to pursuing the appropriate steps of technological readiness as outlined in Figure 2, it is becoming increasingly apparent that “institutional readiness” will be of equal importance in ensuring translation of tissue engineered products into routine clinical practice (10).

**Figure 3:**
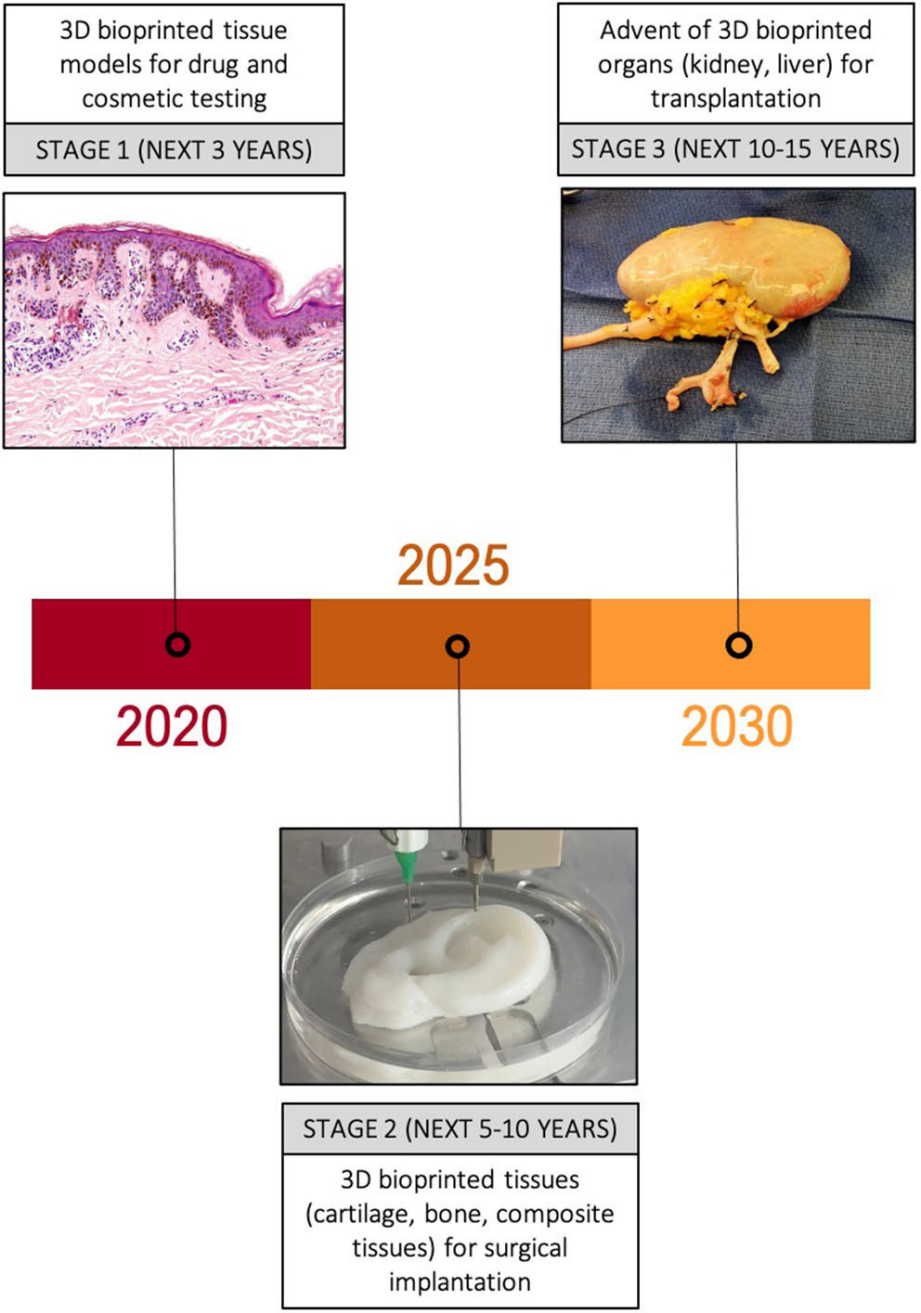
Anticipated trajectory of bioprinting for surgical applications. Major developments in the uses of 3D bioprinted tissue are expected over the next 10–15 years, initially focussing on simple tissue models for drug and cosmetic testing, followed by an increasing number of animal and clinical trials of 3D bioprinted tissue over the next 10 years. Success in these platforms is likely to pave the way for more complex 3D bioprinted constructs such as organs to make an appearance in clinical trials.

The concept of institutional readiness is a social sciences concept which in essence is a “measure of the capacity and willingness of organizations and inter-organizational structures to adopt, respond to and utilize novel technologies” (10). The significance of institutional readiness is that it may impact the technology readiness scale. This may be particularly stark when extrapolated to regenerative medicine interventions: absence of a clear structure within which regenerative medicine interventions can be implemented into health services obstructs the pathway from inception to clinical and commercial success, with the potential to deter investors (10). Institutions who will be responsible for the delivery of regenerative therapies must also display the readiness to cope with the demand for these services. This is likely to include logistical considerations including adequate transport, storage and facilities near to patient for GMP-licensed manufacturing (10). Additionally, facilities will be required for the acquisition of donor and autologous tissue for bioengineering and bioprinting which may include integration with the blood and transplant services as an established clinical body for handling tissue and preparing recipients (36).

One of the keys to translation will be the engagement and active role of clinicians in ensuring the developments from basic science regenerative medicine and bioprinting research is ethically and clinically viable for widespread use in patients. One of the ways in which this might be expedited is through the use of surgically led, rather than research-led units (30).

### What Are the Anticipated Developments in 3D Bioprinting in the Next 20 Years?

As a biofabrication technology, bioprinting encompasses a combination of software, hardware and wetware processes to enable both high throughput and precise placement of cells, biomolecules and biomaterials in a spatially controlled manner. These properties render bioprinting an ideal technology to replicate native living organoids, tissues and organs, “printing a living environment” for both translational medicine and research purposes. It applies the core doctrines of tissue engineering research in which tissue architecture is emulated through the optimal selection of cell, scaffold and growth factor combinations (1), potentiated by the ability to customize, automate and replicate the end tissue engineered product (33).

#### Stage 1: 3D Bioprinting for Drug and Cosmetic Testing

Organovo were the first company to enter the 3D bioprinting sphere in 2007, offering functional bioprinted blood vessels. As early adopters of the technology, the San Diego based biotech paved the way for creation of 3D bioprinted organoids and were long positioned at the head of the market. Their repertoire rapidly expanded to include printed kidney and liver tissue models for research purposes in addition to 3D skin models for cosmetic testing. However, as with many new technological advancements, the need for significant investment front loaded in research and development alongside infrastructure development often creates additional expectation. Whilst overall market investment matched initial hype surrounding the technology, perceived lack of progress resulted in a divestment and directional change for the company in 2019. Twelve years after their 2007 bio-printing debut their CEO Taylor Crouch announced a directional change citing lack of sufficient resources to handle the challenges presented by the “variability of biological performance and related duration of potential benefits” of its lead programme. For many this illustrated one of the key issues in the bioprinting market regarding long term investment balanced against market expectations of a return in a timely fashion. Despite taking a hit in share price, a recent merger with Tarveda Theraputics has seen the company change direction with a focus on precision oncology medicines. In spite of this strategic change, the value of 3D bioprinted models for drug testing and basic science research has significant value and remains a key area of research investment. Currently used cell lines and animal models often fail to emulate the behavior of human tissues and underpin many of the failures of translation to human clinical drug trials. Currently, several drug companies are printing tissue for use in drug testing: Aspect Biosystems have been developing bioprinted lung tissue for this purpose since 2015 (37).

#### Stage 2: 3D Bioprinting of Simple Tissues

It has been predicted that the early stages of using bioprinted products for implantation will occur in the early 2020s (38). It is speculated that the advent of bioprinted implants will spark the emergence of bioprinted tissue for use in regenerative medical and implant-based therapies over the course of the next decade (38). Reconstructive surgery concerns the restoration of form and function to patients affected by congenital abnormalities, trauma, malignancy and burns. In many cases, this population would benefit from relatively small amounts of tissue to restore their form or function. The current treatment options rely heavily on the use of autologous donor tissue to improve the defect. The cost of such a treatment is the creation of a defect or scar elsewhere on the body. Bioprinting offers the potential to evade donor sites and the associated complications of their use with the potential to be life changing. Connective tissues are an achievable medium-term goal for bioprinting. Structures such as cartilage are avascular, aneural and devoid of the extensive cell-cell connections that underpin solid viscera (39). As such, they have been a subject of increasing interest from scientists, clinicians, industry and investors alike (40). Despite global efforts to advance connective tissue bioengineering, there remains a lack of successful translation. There remains a degree of dispute regarding optimal cell sources and scaffolds (41), means of ensuring adequate vascularization (42), characterization and proof of safety prior to implantation (43), and durability in animal models (41). Current high-profile failures in tissue engineering cartilage (in part due to the clinical models in which they were used) highlight the current shortcomings.

#### Stage 3: 3D Bioprinting of Complex Tissues and Organs

The natural progression thereafter would be to engineer composite tissues. Clinically, defects in need of surgical reconstruction often consist of multiple cell types, for example cartilage perichondrium and skin in ear reconstruction (44), or bone, periosteum and mucous membranes in cleft palate repair. Although significant advancements are being made in the engineering of single tissue types, composite tissue engineering adds an additional layer of complexity (45). Production of multilaminar constructs requires a combining of scaffolds, cell source and environments that accommodate each of the intended tissue types. Furthermore, the maintenance of an appropriate interface between the tissue layers, such as in skin, presents a new obstacle, compounded by the need for neovascularisation to enable the survival of the tissue construct (46). Mastery of composite tissue engineering is likely to precede the ability to produce functional organs, which in addition to surmounting the challenges of multiple cell types and vascularisation will require organization of tissue into an organ-specific topography and mimicry of the complex endocrine and physiological roles served by solid organs (47). Furthermore, detailed assessments of the safety, longevity and biocompatibility of smaller bioprinted constructs will need to first be verified and optimized prior to the irreversible action of transplanting a large construct with physiological roles such as a solid organ.

### Is There a Role for 3D Bioprinting in Personalized Medicine?

3D bioprinting offers the opportunity to manage disease through personalized treatments and to produce therapeutics on an industrial scale (48). Bioprinting is likely to augment personalized healthcare through efficient coupling of diagnosis to intervention, translating patient specific images into tailored implants and prosthetics, advancing cell and gene-based therapies and regenerative medicine.

#### 3D Printed Implants and Prosthetics

Firstly, with open source 3D printing files compatible with most printer-based technology available on the internet, there is an increased availability of “blueprints” from which clinicians can choose and initiate printing of a 3D product within minutes (49). By decentralizing the manufacturing process and circumventing transportation and logistical barriers that delay treatment clinicians would have greater access to a range of print files for their patients. For example, upon diagnosis of severe aortic stenosis, the blueprint for a new aortic valve could be downloaded and printed within minutes. Bioprinting also means that the valve could be printed with bioinks such as collagen, that more closely emulate native tissue valves than plastics (50), or even with the patient's native valvular cells to truly personalize the product (51). To a degree, similar processes already exist for dental fillings and neurosurgical cranial plugs (52).

This potential to rapidly download and produce 3D products based on tissue blueprints could even be extrapolated to simple, generic prosthetics. However, the availability of medical images such as CT scans and increasingly seamless integration into 3D printing technologies means true customization and anatomical matching is an achievable reality. In maxillofacial surgery, a combination of contour models, guides, splints and implants have been extensively generated through 3D printing with an average production time of under 24 h (53). This technology could be extrapolated to joint replacements, pacemakers, cochlear implants and other implantable medical devices. Furthermore, three dimensional printing enables the specific seeding of pharmaceutical agents such as antimicrobial, immunomodulatory or analgesic agents during the printing process with the potential to generate a new class of bioactive medical implants (54).

#### Biologically Active 3D Printed Implants

The ability to incorporate cells into the 3D constructs would additionally transform the ability to personalize pharmaceutical and disease management. Currently, several drug companies are printing tissue for use in drug testing: Aspect Biosystems have been developing bioprinted lung tissue for this purpose since 2015 (37). 3D bioprinting research has also been expanded to address the management of diseases such as type 1 diabetes, through the 3D printing of human beta-like cells capable of glucose mediated insulin secretion (55, 56).

The next step in revolutionizing personalized prosthetics is a like-for-like replacement of the defective tissue with tissue engineered constructs. 3D bioprinting offers the ability to print constructs such as ears in the exact shape of the patients missing auricle using a bioink conducive of *de novo* cartilage formation (44). As such, when laced with the patient's own cartilage cells, an exact cartilaginous match of the contralateral ear could be generated. The mastery of 3D bioprinting would merge the accuracy of printed medical prosthetics with the benefits of autologous reconstruction to yield a replacement that would be unparalleled in its resemblance to native tissue (33). As previously discussed, the ultimate potential of 3D bioprinting is the production of patient specific body parts such as organs and limbs, with the capability of revolutionizing personalized medicine and surgery.

### Is 3D Bioprinting the Answer to Organ and Tissue Transplantation?

#### The Clinical Need for 3D Bioprinting

There is an increasing clinical need for organ and tissue replacement therapy (57). In 2016–2017, 6389 patients were awaiting an organ transplant in the UK, yet only 3712 donor transplants were available during this period (58). These statistics underpin the sobering paradigm of modern healthcare: the availability of resources is an inadequate solution to the scale of the clinical problem. In addition to the stark shortage of suitable organ donors, receipt of an organ transplant requires a lifetime of immunosuppressive medication whilst still retaining a lifetime risk of rejection and immune-mediated diseases. Ultimately, these patients may find themselves in need of an additional organ transplant or even facing death. The promise of bioprinting is to truly personalize tissue engineering: using a patient's own cells and genetic material to generate a replacement viscera in a shape and structure that matches their own anatomy. Biologically, this obviates the need for HLA matching, the risk of acute rejection and facilitates long-term integration of the organ into the recipient. Structurally, it enables the combination of multiple tissue types arranged precisely in the tissue's native microarchitecture and microenvironment to provide an organ that is truly the patient's own.

#### The Role of Organ Biofabrication

Despite *in vitro* and *in vivo* studies supporting the feasibility of tissue engineering for use in a multitude of clinical scenarios (59, 60) obstacles remain that are hindering clinical translation. At the top end of the spectrum, solid organs such as the liver display a complex three-dimensional array of different tissue types that work in synergy to maintain the structural and functional components of the organ. The complex interplay between bile ducts, hepatocytes, vasculature and connective tissue act synergistically to serve digestive, endocrine and hematological roles. Although tissue engineering and 3D bioprinting may ultimately be able to emulate the complex topography and function of solid viscera, replication of this interplay *in vitro* will be fraught with technical and biological difficulty (61).

The high resolution of bioprinting conveys the advantage of enabling the deposition in nano to microscale array to mirror histological and macroscopic morphology of different tissues (62). In the post-processing phase, bioreactors offer a dynamic environment for tissue maturation to occur, though precautions must be implemented to minimize the risk of tissue damage during the maturation process (63). Bioprinting is currently the best suited biofabrication method to achieve the required porosity, geometry and interconnectivity of complex structures such as solid viscera and is likely to underpin major advancements in the field over the next 20 years. To date, there have been a number of first in human implantations of tissue-engineered constructs, not all of which have been successful, highlighting the need for robust preclinical evidence and high-quality clinical trials in this field to ensure patient safety (Table 1).

**Table 1:** Applications of tissue-engineered constructs in humans [adapted from Al-Himdani et al. (44)].

**Organ/tissue**	**No. of patients**	**Cell source**	**Outcomes**	**References**
Bladder	7	Bladder urothelial and muscle cells	Improved volume and compliance with no metabolic consequences at mean 46 months follow-up	(64)
Trachea	1	Recipient MSCs	Functional airway with a normal appearance and mechanical properties at 4 months, recent controversy	(29)
Urethra	5	Muscle and epithelial cells	Maintenance of wide urethral calibers without strictures, normal architecture on biopsy at 3 months following implantation	(65)
Nasal cartilage	5	Autologous nasal chondrocytes	Good structural stability and respiratory function after 1 year	(66)
Vaginal organs	4	Vulval biopsy—epithelial and muscle cells	Tri-layered structure on biopsy with phenotypically normal smooth muscle and epithelia with follow-up up to 8 years	(67)
Auricular cartilage	5	Autologous auricular chondrocytes	Evidence of cartilage formation at 6 months in 80% of patients, structural deformation noted in most cases	(35)

### The Role and Growth of the Global 3D Bioprinting Market

#### Market Overview: Base Technology

The exponential development of the 3D bioprinting market in both the academic and commercial settings is largely due to the successful development and adaptation of the base technologies first pioneered in the 1980s. The 3D printing and additive manufacturing (AM) industry surpassed forecasted growth expectations and in the “2018 Wohlers Report” they cited a 21% growth of AM products and services world-wide, currently valuing the market at $7.336 Billion, $1 Billion ahead of initial projections. Key investments in Research and Development and the significant development of entry level platform technologies saw an estimated 520,000 desktop 3D printers under $5000 sold in just 2 years, with Ultimaker, MakerBot and Aleph Objects contributing to an estimated $500 million made from just this technology alone (68). Regardless of the product offering companies continue to use and refine one of the four main print-head technologies; extrusion, laser-induced, inkjet and microvalve. Extrusion (syringe based) technology continues to command the greatest market share due to its affordability, ease of adaptation and broad applications.

#### Bioprinting Market

There are numerous market forces which have been instrumental in driving up the compound annual growth rate (CAGR) with global market projections of between 26 and 36% in the next 5–10 years (69). Advancements in production technology and biomaterials innovation alongside increasing cost and regulatory complexity in the manufacture and testing of pharmaceuticals and cosmetics saw the first wave of companies enter the market in 2014 offering novel printers and tissue specific assays. As the market develops there appears to be a divergence with companies such as EnvisionTec, GeSIM, Cellink, and BioBots choosing to focus on printer technology and associated consumables, whereas Organovo focus on the production of cellular products and tissue assays. Maintaining its position as the market leader and first ever publicly traded bioprinting company; Organovo initially offered a small number of human tissue assays including liver and kidney for drug testing and pre-clinical research and has since developed a custom tissue partnership offering the opportunity to develop unique tissue models and assays specific to an individual company or researchers needs. In 2014 Organovo in partnership with L'Oreal, developed 3D bioprinted skin models to get ahead of the EU directives banning the testing of cosmetics on animals (13).

#### Future Developments

The creation of complex human tissue arrays and organoids has not gone unnoticed by researchers around the world looking to better create 3D models of complex diseases such as cancer. The production of 3D vascularised tumor models “organ on a chip” has been created to better understand the complex interplay between cancer and multi-organ metastasis and paracrine signaling mechanisms in the regulation of breast cancer metastasis (70). This novel utilization of 3D printing has the potential to advance our understanding of complex disease and develop novel personalized treatments for diseases such as caner which currently account for one in seven deaths worldwide (71). The continued development and application of this base technology promises to 1 day make the creation of bespoke tissue engineered constructs and “made to order” solid complex organs a reality. The technological revolution in the last two decades has seen the development of intelligent bio-inks, refinement of printing techniques and production of novel biomaterials to facilitate the creation of custom scaffolds to support cellular growth (72). Since 2014 a number of 3D bioprinting companies, start-ups and R&D spinouts have entered the market contributing to the commercial development of this novel technology and creating a projected market value based on the early success and novel application of 3D bioprinted products (38). With a market value estimated at around $680 Million in 2016, industry reports project growth to reach $1.9 Billion by 2027 (73).

## Conclusions

3D printing and bioprinting has the potential to be the single biggest technological disruptor to the current model for design and delivery of healthcare and research in this century. The incorporation of human cells and biocompatible materials into 3D printing practice is set to deliver a paradigm shift in the application of 3D printing for surgery, offering the potential to 3D print living tissue and organs. The promise to 3D print *de novo* body parts, obviate the need for organ transplantation and to replace the role of animals in the development and testing of novel drugs, means patients could potentially have access to a bespoke treatments at every point in their healthcare journey.

The diverse applications of bioprinting technology have already been demonstrated on a global scale, leading to the production of novel constructs from vessels and composite tissue, to organoids and complex cellular and tissue models for drug, cosmetic and experimental testing. The 3D bioprinting market has seen off shoot companies set up to corner a specific sub-set of the production and manufacturing of complex 3D printed tissues; from desktop 3D bioprinters and bioinks, to scaffolds pre-loaded with and without growth factors generating a market value in the $US billions. The diversification of this technology and its associated components demonstrate the key issue with this extraordinary technology and potential difficulty in harnessing its true potential; the lack of “end to end” visibility by any one agency.

The translation of 3D printed constructs into clinical practice is challenging. The optimisation of the translational pathway demands concerted efforts from scientists, engineers and clinicians, contextualized within an infrastructure in which an effective supply chain exists. It is no longer sufficient for scientists, clinicians and regulatory bodies to exist in operational silos: there is a need for a collaborative effort to translate this impactful technology into a real-world healthcare setting. In order to harness the true potential of 3D printing in surgery, surgeons will need to keep abreast of developments in the field, identify niches in which this technology can be applied and encourage its integration into mainstream surgical practice. With incremental advances in 3D printing and bioprinting expected over the next century, the impact on the future of surgery could be transformational.

## Author Contributions

IW, ZJ, and TJ conceived the idea for the manuscript. TJ and EC drafted the manuscript with significant contribution from ZJ and IW. All authors contributed to the article and approved the submitted version.

## Conflict of Interest

The authors declare that the research was conducted in the absence of any commercial or financial relationships that could be construed as a potential conflict of interest.
